# Dengue Virus Type 4, Manaus, Brazil

**DOI:** 10.3201/eid1404.071185

**Published:** 2008-04

**Authors:** Regina Maria Pinto de Figueiredo, Felipe Gomes Naveca, Michele de Souza Bastos, Miriam do Nascimento Melo, Suziane de Souza Viana, Maria Paula Gomes Mourão, Cristóvão Alves Costa, Izeni Pires Farias

**Affiliations:** * Fundação de Medicina Tropical do Amazonas, Manaus, Brazil; † Fundação Alfredo da Matta, Manaus, Brazil; ‡ Instituto Nacional de Pesquisas da Amazônia, Manaus, Brazil; § Universidade Federal do Amazonas, Manaus, Brazil; 1 These authors contributed equally to this work.

## Abstract

We report dengue virus type 4 (DENV-4) in Amazonas, Brazil. This virus was isolated from serum samples of 3 patients treated at a tropical medicine reference center in Manaus. All 3 cases were confirmed by serologic and molecular tests; 1 patient was co-infected with DENV-3 and DENV-4.

Dengue fever is the main arthropod-borne viral disease of humans and a resurgent global public health concern; an estimated 50–100 million cases occur every year, primarily in the tropical regions of the world (*1**–**3*). Dengue viruses (DENVs) belong to the genus *Flavivirus*, family *Flaviviridae*. They are single-stranded, positive-sense, RNA viruses grouped into 4 antigenically related, but distinct, serotypes (DENV-1 to DENV-4) (*1*).

DENV infection has increased in Brazil in the past decade, particularly after 1994, as a result of *Aedes aegypti* dissemination. Vector dispersion was followed by introduction of DENV-1, DENV-2, and DENV-3 in major Brazilian cities. Co-circulation of different serotypes has caused cases of the more severe forms of dengue, namely, dengue hemorrhagic fever (DHF) and dengue shock syndrome (DSS) (*4*). Two dengue epidemics occurred in Manaus (3°5′S, 60°W), the capital of the state of Amazonas (Figure), during 1998–1999 and in 2001 (*5**,**6*). DHF cases were observed in association with DENV-1 and DENV-2 in the most recent epidemic. Currently, DENV-3 also co-circulates in Manaus (*7*).

**Figure:**
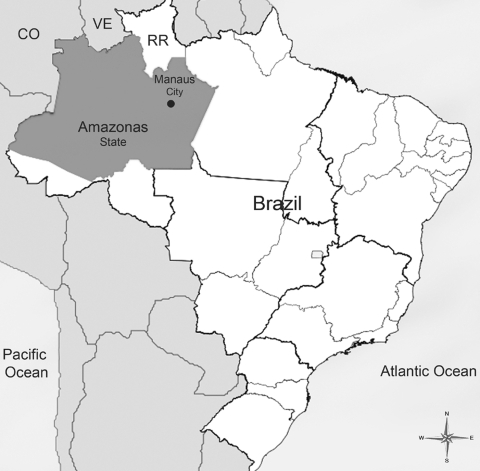
Location of Amazonas State and Manaus City, Brazil. CO, Colombia; VE, Venezuela; RR, State of Roraima.

## The Study

A study on serologic and molecular characterization of DENV isolates was initiated in January 2005 at the Fundação de Medicina Tropical do Amazonas (FMTAM). Parasite-negative patients who had clinical symptoms of malaria were invited to participate in the study. Each patient received essential information and signed a consent form approved by the FMTAM ethical committee.

All serum samples were collected during the acute phase of illness and tested for DENV infection by using 3 methods. The first method was virus culture, for which serum samples were placed on the *Aedes albopictus* cell line C6/36 grown in Leibovitz-15 medium containing 5% fetal bovine serum, followed by viral antigen identification with type-specific monoclonal antibodies in an indirect immunofluorescence assay (*8*). The second method was detection of immunoglobulin M antibodies to DENV by an ELISA on serum samples from patients >7 days after onset of symptoms (*9*). The third method was nucleic acid amplification and typing by a seminested reverse transcription–PCR (RT-PCR) protocol on the basis of that described by Lanciotti et al. (*10*).

Briefly, viral RNA was extracted by using the QIAamp Viral RNA Mini Kit (QIAGEN, Valencia, CA, USA), and reverse transcription was conducted on 5 μL of extracted RNA with Superscript III (Invitrogen, Carlsbad, CA, USA) and random primers. After incubation for 1 h at 50°C, 2 μL of each cDNA was subjected to PCR amplification with D1 and D2 primers for 35 cycles consisting of 1 min at 94°C, 1 min at 55°C, and 1 min at 72°C, and a final extension for 10 min at 72°C. A second round of amplification was conducted with 10 μL (diluted 1:100) of the first amplicon, a mixture of type-specific reverse primers (TS1–TS4), and the conserved forward primer D1. The same cycling parameters were used as in the first reaction.

DENV-4 was detected in 3 samples (AM750, AM1041, and AM1619) by virus culture or RT-PCR. It was identified as a co-infecting virus with DENV-3 in isolate AM750; samples AM1041 and AM1619 represented single DENV-4 infections (Table). To confirm these results, samples were reamplified with each PCR typing primer separately. Generated amplicons were cloned into a TA vector (Invitrogen), and >3 colonies for each sample were sequenced in both directions by using the BigDye Terminator Cycle Sequence Kit (Applied Biosystems, Foster City, CA, USA). DENV-3 and DENV-4 nucleotide sequences obtained were subjected to a basic local alignment search tool (BLAST) (www.ncbi.nlm.nih.gov/) analysis that used the megablast algorithm optimized for highly similar sequences. Using this approach, we obtained sequences with identities ranging from 95% to 99% for DENV-3 and 94% to 98% for DENV-4 for isolates AM750-D3, AM750-D4, and AM1619. These results confirmed our results obtained with monoclonal antibodies and PCR typing assays. The nucleotide sequences were deposited in GenBank under accession nos. EU127898 (AM750-D3), EU127899 (AM750-D4), and EU127900 (AM1619).

**Table:** Results of different methods used to confirm dengue virus type 4 (DENV-4) infection, Manaus, Amazonas, Brazil*

Isolate	IgM antibody capture ELISA	Virus culture†					M-PCR					S-PCR				BLAST
		D1	D2	D3	D4		D1	D2	D3	D4		D1	D2	D3	D4	
AM750	–	–	–	+	+		–	–	+	+		–	–	+	+	DENV-3/ DENV-4‡
AM1041	+	–	–	–	+		–	–	–	+		–	–	–	+	NS
AM1619	+	–	–	–	+		–	–	–	+		–	–	–	+	DENV-4

*IgM, immunoglobulin M; M-PCR, multiplex typing PCR described by Lanciotti et al. (*10*); S-PCR, single serotype-specific primer PCR; D, dengue serotype; BLAST, basic local alignment search tool; NS, not sequenced. †Viral antigens detected by immunofluorescence with type-specific monoclonal antibodies. ‡Isolate AM750 is from a person with a co-infection; different clones were sequenced.

The 3 DENV-4–positive samples were obtained from patients who lived and worked in Manaus and reported no travel history for >15 days before onset of symptoms. These samples were obtained during another study that identified 62 DENV-positive samples from January 2005 through June 2007 (24 DENV-2, 35 DENV-3, and the 3 DENV-4 cases in our study) among 128 samples tested from patients in 14 municipalities in Amazonas.

## Conclusions

Since its introduction into the Western Hemisphere in 1981, DENV-4 has been associated with dengue fever and only sporadically associated with serious cases of DHF or DSS (*1*). A study in Colombia found more DHF patients infected with DENV-2 than with DENV-3 or DENV-4 (*11*). Conversely, another study showed an association of DENV-4 with an epidemic of DHF that occurred in Mexico in 1984 (*12*).

There are many host (and perhaps viral) factors in dengue infections that may lead to development of DHF. On the basis of the antibody-dependent enhancement hypothesis, the most important factors would be those generated by the patient’s immune response upon secondary infections (*13*). The 3 isolates reported in our study were from patients with no travel history, which indicates that DENV-4 is present in Manaus. Detection of DENV-4 in Brazil co-circulating with other DENV serotypes endemic to this country represents an increased risk for DHF or DSS because many persons have been sensitized by previous dengue infections but are not protected against infection with DENV-4.

The first report of DENV-4 in Brazil was in the state of Roraima in 1982. Since that time, no other isolate of DENV-4 has been reported in any part of Brazil (*14**,**15*). The resurgence of DENV-4 in the Amazon region of Brazil most likely resulted from the proximity of Brazil to DENV-4–endemic countries (Venezuela and Colombia). Additional genotyping studies are being conducted to verify this assumption and to obtain information on dengue epidemiology in Brazil.

Our study documents the detection of DENV-4 in Manaus, Amazonas, and the first isolation of this serotype in Brazil in 25 years. These findings reinforce the need for continual epidemiologic studies and use of classic and molecular approaches in the surveillance of emerging or reemerging diseases.
